# In-Plane Ultrasound-Guided Botulinum Toxin Injection to Lumbrical and Interosseus Upper Limb Muscles: Technical Report

**DOI:** 10.7759/cureus.45073

**Published:** 2023-09-12

**Authors:** Alexandros Toliopoulos

**Affiliations:** 1 Neurology, Rehabilitation and Sports Medicine Clinic Evosmos, Thessaloniki, GRC; 2 Sports Medicine, Rehabilitation and Sports Medicine Clinic Evosmos, Thessaloniki, GRC

**Keywords:** ultrasound, botulinumtoxin, botulinum, in-plane, interossei, lumbricals

## Abstract

This technical report presents an innovative approach utilizing in-plane ultrasound-guided injections to precisely target the lumbrical and interosseous muscles within the upper limb. The report also elucidates the rationale and advantages of incorporating ultrasound guidance in the administration of Botulinum toxin (BTX) injections, encompassing the real-time visualization of needle trajectory and accurate localization of the target muscle groups while delving into the pertinent anatomical details of these muscles and their significance in diverse neuromuscular conditions.

The step-by-step procedure for conducting in-plane ultrasound-guided BTX injections to the lumbrical and interosseous muscles is delineated, emphasizing critical technical considerations and potential pitfalls to be vigilant of during the procedure. Furthermore, the article addresses the significance of selecting appropriate BTX dosages and injection sites based on individual patient presentations and clinical indications.

Overall, the in-plane ultrasound-guided BTX injection technique presents a promising approach for providing precise and targeted treatment to the lumbrical and interosseous muscles in the upper limb. It offers clinicians an alternative to injections performed without guidance or out-of-plane ultrasound-guided injections, potentially decreasing the likelihood of complications and enhancing treatment outcomes for patients with a range of neuromuscular conditions. However, further research and comparative studies are necessary to establish the long-term efficacy and safety of this technique, thus confirming its role in clinical practice.

## Introduction

Botulinum toxin (BTX) injections have revolutionized the management of upper-limb neuromuscular disorders. Nowadays, they are the first-line option for patients suffering from focal spasticity and dystonia, providing significant relief [1-3]. These conditions can stem from various etiologies, including stroke, cerebral palsy, and other neurological disorders, resulting in debilitating functional impairments and diminished quality of life [2].

Traditionally, BTX injections were administered using a blind technique or an out-of-plane ultrasound-guided injection [1,3], relying solely on anatomical landmarks, palpation, or transverse ultrasound images to identify the target muscles. However, these approaches possess limitations that can lead to inaccurate needle placement and suboptimal treatment outcomes. Moreover, the absence of real-time visualization during the injection procedure increases the risk of adverse effects and potentially compromises patient safety [4,5].

In-plane ultrasound guidance has the potential to revolutionize precision and safety in BTX injections for the lumbrical and interosseous muscles of the upper limb. Ultrasound imaging facilitates real-time visualization of the musculoskeletal structures, enabling clinicians to accurately pinpoint the target muscles, surrounding neurovascular structures, and any patient-specific anatomical variations [1,4].

This article aims to deliver a comprehensive overview of the in-plane ultrasound-guided BTX injection technique for the lumbrical and interosseous muscles in the upper limb. It will cover the pertinent anatomy of these muscles, their role in various neuromuscular conditions, and the significance of accurate targeting to attain favorable therapeutic outcomes.

Additionally, this article will explore the technical aspects of in-plane ultrasound-guided injections, including the necessary equipment, patient positioning, and a step-by-step procedural guide. It will also address potential challenges and pitfalls associated with this technique, focusing on strategies for mitigation.

Finally, an examination of existing clinical evidence and outcomes associated with in-plane ultrasound-guided BTX injections will be presented, spotlighting the benefits of this technique and its potential to reshape the management of upper limb neuromuscular disorders.

As far as the authors are aware, this article stands as one of the earliest and most comprehensive works to detail this injection technique. The sole other reference is authored by Ata et al. in 2023 [6]. By delving into the intricacies of this technique, it is anticipated that its role as a valuable addition to the array of treatments available for patients with upper-limb neuromuscular disorders will be further solidified.

## Technical report

The novel in-plane ultrasound-guided technique

Ultrasound Equipment, Settings, Patient Positioning, and Probe Placement

An ultrasound machine equipped with a high-frequency linear probe offers exceptional resolution and visualization of superficial structures, rendering it well-suited for guiding injections into small and intricate muscles. In the case of the hand, a high-frequency range of 9-15 MHz is typically deemed appropriate [2]. Higher frequencies provide superior resolution for superficial structures, enabling clear visualization of the target muscles and adjacent tissues. The depth setting should be configured to capture the target muscles effectively while avoiding excessive penetration into surrounding tissues. Typically, a 27-gauge needle with a 3-4 cm length is employed [2,4].

The patient's hand should be positioned comfortably, ensuring unrestricted access to the injection site. The ultrasound probe is positioned over the dorsum of the hand, aligned parallel to the target muscles in a longitudinal view. Precise adjustments are made to the probe's orientation to obtain optimal images of the lumbrical and interosseous muscles in the second to fifth dorsal metacarpal areas [6].

Identification of Lumbrical and Interosseous Muscles, Needle Insertion, and Botulinum Toxin Injection

The ultrasound probe is aligned parallel to the hand. The lumbrical muscles manifest as hypoechoic bands situated on the palmar aspect of the fingers, whereas the interosseous muscles are visualized as hypoechoic structures positioned between the metacarpal bones. By discerning these distinctive muscle patterns, the practitioner can accurately guide the needle into the target muscles. Needle insertion and toxin injection are meticulously executed under real-time visualization [6,7].

**Figure 1:**
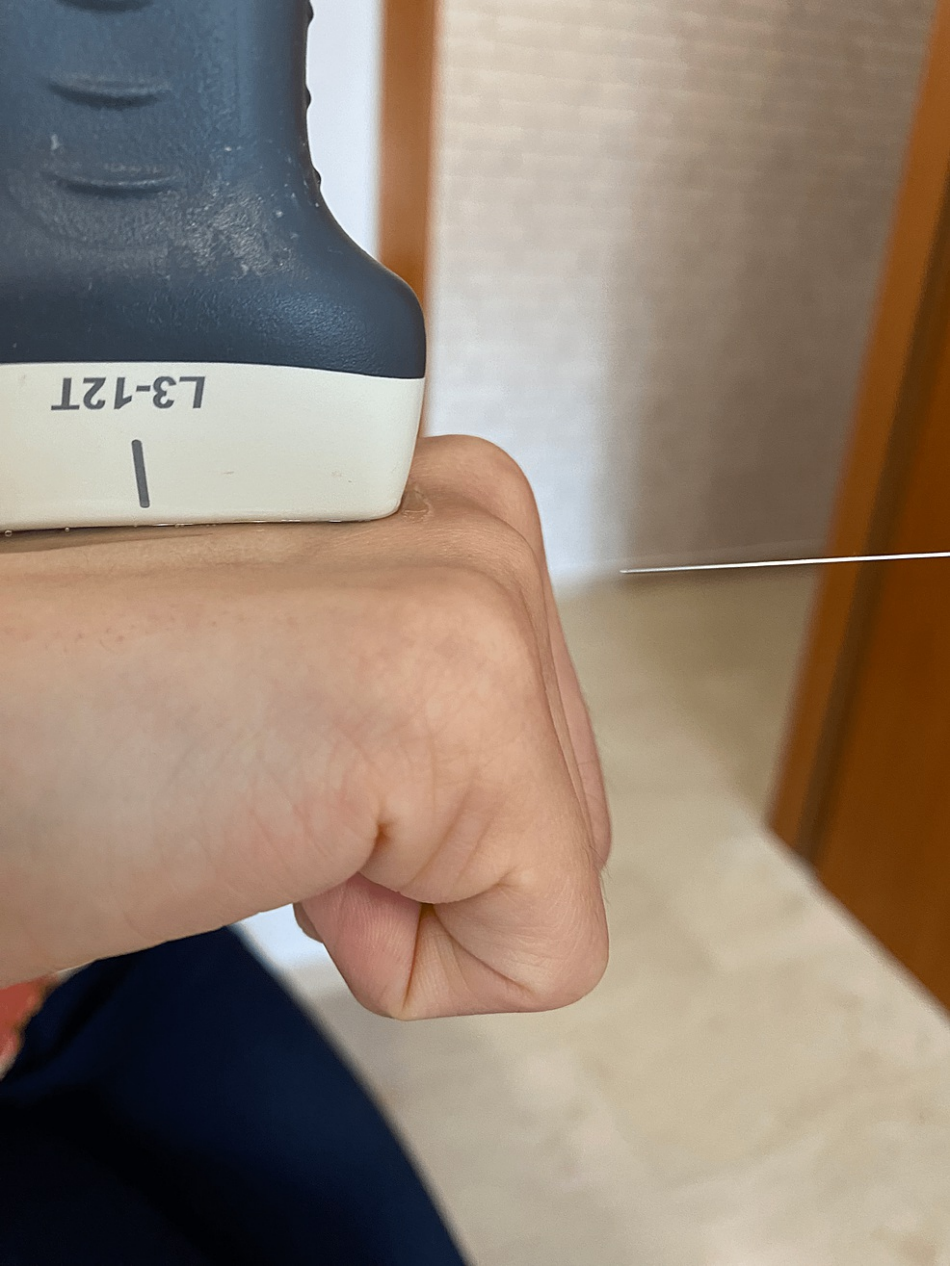
In-lane US-guided injection. Insertion point of the needle. This is the insertion point for the needle in an in-plane ultrasound-guided injection technique for a patient with upper limb spasticity [6]. The insertion point was selected to ensure a parallel ultrasound view of the needle.

**Figure 2:**
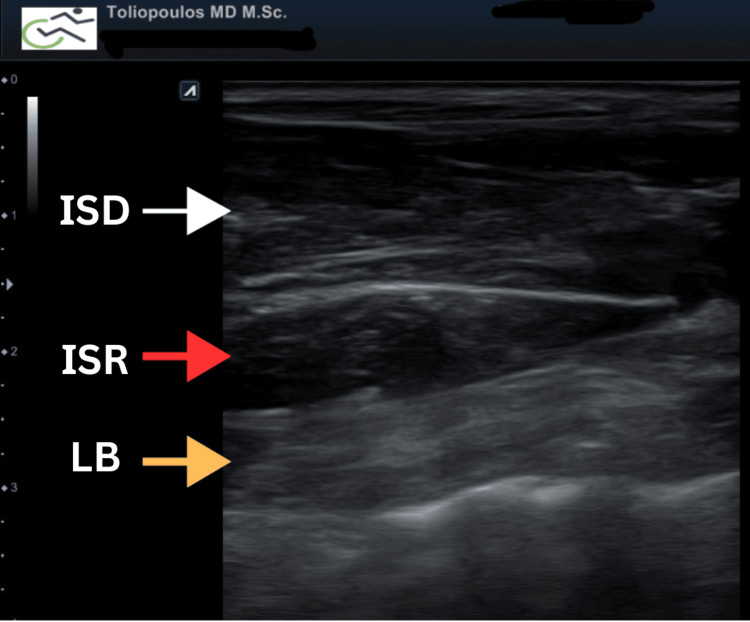
Ultrasound appearance of the hand intrinsic muscles. The white arrow indicates the dorsal interosseous muscle, the red arrow points to the palmar interosseous muscle and the yellow arrow highlights the lumbrical muscle.

The insertion point is distal to the metacarpophalangeal (MCP) joint, within flexed fingers. Once appropriately positioned within the target muscle, the botulinum toxin is administered slowly and uniformly. This approach enhances the even distribution of the toxin, improving treatment effectiveness and reducing the risk of complications. Ultimately, these measures lead to enhanced outcomes for patients dealing with conditions like spasticity or hand dysfunction [6] (Figures 1-2 and Videos 1-2).

**Video 1:** Ultrasound appearance of the 2nd-3rd dorsal metacarpal space. The video clearly shows the interosseous and lumbrical muscles at the 2nd to 3rd metacarpal space of the upper limb. From dorsal to palmar, the muscles are as follows: (1) dorsal interosseous, (2) palmar interosseous, (3) lumbrical muscles.

**Video 2:** In-Plane ultrasound guided BoNT technique at lumbricalls and interosseous upper limb muscles. This video demonstrates the in-plane ultrasound-guided botulinum toxin technique on the lumbrical and interosseous muscles of the upper limb. The white arrow indicates the dorsal interosseous muscle, the red arrow points to the palmar interosseous muscle, the yellow arrow highlights the lumbrical muscle, and the blue arrow indicates the needle insertion point. The injected toxin appears hypoechoic.

Comparative analysis

Differences Between the In-Plane and Out-of-Plane Techniques for Botulinum Toxin Ultrasound-Guided Injections

The selection between the in-plane and out-of-plane techniques hinges on the physician's familiarity and experience with both methodologies, along with the patient's individual anatomical considerations and preferences. These techniques can be performed on most muscles in the body, and the main difference between them is the insertion point of the needle. Physicians should evaluate the merits and drawbacks of each technique to determine the most suitable approach for specific cases. Both techniques are applicable to the lumbrical and interosseous muscles of the hand [2,4].

Several advantages are associated with the in-plane injection technique, including real-time visualization of the needle pathway, reduced risk of inaccurate injections, improved patient outcomes, and enhanced patient satisfaction with the procedure [7].

Conversely, the in-plane technique might demand greater skill and experience to be proficiently executed, particularly for smaller and deeper muscles. The out-of-plane technique is comparatively simpler to perform, which can be advantageous for practitioners with less experience or when treating multiple muscles [7].

Advantages Over Other Imaging Modalities and Limitations

Unlike fluoroscopy or MRI, ultrasound is readily accessible, cost-effective, and does not subject patients to ionizing radiation. In contrast to EMG guidance, ultrasound offers real-time visualization of the needle trajectory, ensuring precise targeting without necessitating additional invasive procedures. The in-plane technique using ultrasound permits real-time adjustments during the injection process, augmenting procedural control and safety. Its portability and convenience render it a preferred choice, providing heightened accuracy, improved outcomes, and patient satisfaction in botulinum toxin injections for the lumbrical and interosseous muscles [3,8].

The primary limitation of the technique lies in its dependence on the practitioner's skill. Proficiency in the ultrasound-guided in-plane technique for lumbrical and interosseous injections demands a learning curve and specialized training [7,9]. Healthcare providers must attain competence in ultrasound imaging and needle guidance to ensure precise needle placement and effective toxin administration [10].

## Discussion

The lumbrical muscles are a group of four small intrinsic hand muscles situated on the palmar side of the hand, spanning the I-V phalanges. They originate from the tendons of the flexor digitorum profundus (FDP) muscle within each finger and course along the palmar aspect of the hand [11,12]. Subsequently, these muscles loop dorsally around their respective MCP joints, eventually inserting into the extensor hood mechanism of each finger. This distinctive configuration empowers the lumbricals to function as both extensors at the MCP joint and flexors at the proximal and distal interphalangeal (PIP and DIP) joints [11,12].

The interosseous muscles are categorized into two groups: the palmar interossei (comprising three muscles) and the dorsal interossei (comprising four muscles). These muscles originate from the metacarpals and insert onto the proximal phalanges of the fingers [Ikebuchi Jang].

The lumbrical and interossei muscles serve pivotal functions in upper limb activity, particularly in hand movements, grip strength, precision gripping, finger flexion and extension, stabilization of the hand's arch, and fine motor control [11,12].

Traditional methods of administering botulinum toxin injections, without the aid of ultrasound guidance, have been employed in clinical practice. Although these approaches have demonstrated efficacy, they are not devoid of limitations and potential complications [10]. It is imperative to recognize these factors when conducting botulinum toxin injections.

Conventionally, botulinum toxin injections are administered based on anatomical landmarks and by palpating the target muscles. However, this technique can be susceptible to variability and imprecision due to individual anatomical differences and challenges in accurately delineating muscle boundaries [10]. Consequently, there exists an elevated risk of inaccurate needle placement and suboptimal delivery of the toxin [10]. In conventional techniques, real-time visualization of the needle and target structures is lacking during the injection process, which can contribute to increased discomfort for the patient. Furthermore, potential complications, such as hematomas and nerve injuries, can arise [13].

Out-of-plane ultrasound-guided BTX injection techniques come with particular limitations that may result in complications. Some of these limitations encompass suboptimal needle visualization when compared to in-plane techniques, making it demanding to ensure precise needle placement and potentially heightening the likelihood of inadvertent injury to adjacent structures. On occasion, due to limited visualization, multiple needle passes may be necessary to reach the target muscle, extending the procedural time and causing discomfort to the patient. Challenges in visualizing the needle pathway could lead to uneven distribution of the BTX dosage within the target muscles, potentially resulting in inconsistent treatment outcomes [1].

The in-plane ultrasound-guided technique for BTX treatment of the interosseous and lumbrical muscles in the upper limb could potentially result in significant functional improvements [14,15]. These improvements may include reduced pain, increased range of motion, and enhanced hand function, which could endure over an extended follow-up period.

The real-time visualization provided by the in-plane technique presents a critical advantage in ensuring accurate needle placement within the target muscles. This aspect of the technique reduces the risk of off-target injections, thereby minimizing the potential for adverse events and optimizing the effectiveness of the treatment. The capability to continuously visualize the needle tip during the procedure provides a level of safety that is not easily achievable with other injection methods [13].

Furthermore, the in-plane technique can be associated with enhanced safety. By mitigating the risk of inadvertent injury to nearby structures, such as blood vessels and nerves, the in-plane approach solidifies its suitability for use in delicate anatomical regions, like the upper limb.

Nonetheless, this technique does come with limitations. It is crucial to consider the potential learning curve associated with the in-plane technique. Proficiency in musculoskeletal ultrasound is essential for mastering this approach, as it demands skilled hand-eye coordination and familiarity with the anatomy of the upper limb muscles.

## Conclusions

The in-plane ultrasound-guided technique for injecting botulinum toxin into the lumbrical and interosseous muscles offers a promising solution for managing spasticity and dystonia in the upper limb. Despite recognizing the possible learning curve associated with this approach, its enhanced precision, safety, and efficacy hold the potential to significantly enhance patient outcomes and minimize complications. Further research studies and the widespread adoption of this technique in clinical practice may further establish its value as an indispensable tool in the realm of neuromuscular medicine.
